# Comparison of the analgesic effects of liposomal bupivacaine in ultrasound-guided erector spinae plane block and surgeon-administered intercostal nerve block following video-assisted thoracoscopic lung resection: protocol for a randomized controlled trial

**DOI:** 10.3389/fmed.2025.1564738

**Published:** 2025-03-31

**Authors:** Jing Yan, Yu-shu Feng, Xiao-yan Zheng, Yang Zhang, Hua-yue Liu, Yu-fan Yang, Jing-jie Wan, Ke Peng, Hong Liu, Fu-hai Ji, Xi-sheng Shan

**Affiliations:** ^1^Department of Anesthesiology, First Affiliated Hospital of Soochow University, Suzhou, Jiangsu, China; ^2^Institute of Anesthesiology, Soochow University, Suzhou, Jiangsu, China; ^3^Suzhou Medical College, Soochow University, Suzhou, Jiangsu, China; ^4^Ambulatory Surgery Centre, First Affiliated Hospital of Soochow University, Suzhou, Jiangsu, China; ^5^Department of Anesthesiology and Pain Medicine, University of California, Davis, Sacramento, CA, United States

**Keywords:** erector spinae plane block, intercostal nerve block, liposomal bupivacaine, video-assisted thoracoscopic surgery, postoperative pain

## Abstract

**Background:**

The analgesic efficacy of liposomal bupivacaine (LB) for ultrasound-guided erector spinae plane block (ESPB) and thoracoscopic intercostal nerve block (ICNB) in thoracic surgery remains uncertain. This study aims to evaluate the analgesic efficacy of anesthesiologist-performed ESPB using LB versus surgeon-administrated ICNB with LB in patients undergoing video-assisted thoracoscopic surgery (VATS) lung resection.

**Methods:**

This single-center, prospective, randomized, double-blinded trial will include 120 adult patients scheduled for VATS lung resection. Patients will be randomly assigned 1:1 to the ESPB group or ICNB group. Each patient will receive either an ESPB or ICNB at the end of the surgery, along with patients-controlled intravenous analgesia (PCIA) as part of a postoperative multimodal analgesia. The primary outcome is the average numeric rating scale (NRS) pain scores at rest over 72 h postoperatively (average of three 24-h time points: 24, 48, and 72 h). Secondary outcomes include NRS pain scores at rest and during activity at 24, 48, and 72 h postoperatively, Quality of Recovery 15 scores at 24, 48, and 72 h postoperatively, time to first press on the PCIA device, total opioid consumption within 72 h postoperatively, time to initiate independent bedside mobilization, length of postoperative hospital stay, and the incidence of chronic pain (defined as an NRS score ≥ 1) at 3 months post-surgery. Analyses will be performed in the modified intention-to-treat population.

**Discussion:**

We hypothesize that anesthesiologist-performed ultrasound-guided ESPB with liposomal bupivacaine will result in lower average numeric rating scale pain scores over 72 h compared to surgeon-administrated thoracoscopic ICNB in patients undergoing VATS lung resection. The findings of this study aim to provide evidence to optimize postoperative analgesic regimens for patients undergoing VATS lung resection.

**Clinical trial registration:**

http://www.chictr.org.cn, identifier ChiCTR2400092927.

## Introduction

Minimally invasive thoracic surgery such as video-assisted thoracoscopic surgery (VATS) has become the predominant approach for lung resection over the past decade (1). Despite its minimally invasive nature, VATS can still cause significant pain in 25–44% of patients, with some experiencing chronic pain lasting for months or even years, profoundly impacting their quality of life (2–4). Consequently, multimodal analgesia, particularly regional nerve block techniques, has become essential for patients undergoing VATS lung resection. However, the effectiveness of these techniques is often limited by the relatively short duration of standard local anesthetics, which typically last less than 24 h. Liposomal bupivacaine (LB) is a long-acting local anesthetic encapsulating water-soluble bupivacaine within liposomes, providing sustained drug release for 72–96 h (5). Initially approved by the U.S. Food and Drug Administration in 2011 for single-dose wound infiltration to manage postsurgical analgesia in adults, its use has since expanded to include fascial plane blocks, showcasing safety in various clinical settings (6, 7).

LB is frequently employed as postoperative analgesia for intercostal nerve blocks (ICNB) in thoracic surgery, and ICNB can be easily performed by surgeons during VATS (8, 9). Due to the limited specific dermatomal distribution of its analgesic effect, the ability of ICNB to provide superior pain control following thoracoscopic procedures remains controversial (10, 11). The erector spinae plane block (ESPB), first introduced in 2016 (12), has emerged as a promising interfascial plane block technique for thoracic pain management, known for its simplicity, high safety profile, and reliable analgesic effectiveness (13, 14). Recent studies comparing ESPB and ICNB using plain local anesthetics in thoracic surgery have yielded inconsistent results. One study demonstrated that ESPB with plain local anesthetics provides superior analgesia and reduces perioperative analgesic requirements compared to ICNB in patients undergoing mini-thoracotomy (15). Another study reported comparable postoperative analgesic effects between the two groups in patients undergoing VATS lung resection (16). To date, no prospective studies have compared the analgesic efficacy of LB when used in anesthesiologist-performed ultrasound-guided ESPB versus surgeon-administrated thoracoscopic ICNB in thoracic surgery.

Therefore, we designed this randomized controlled trial to evaluate the effects of LB when used in these two techniques on postoperative pain in patients undergoing VATS lung resection. We hypothesized that patients receiving anesthesiologist-performed ultrasound-guided ESPB with LB would have lower average numeric rating scale pain scores over 72 h compared to those receiving surgeon-administered thoracoscopic ICNB.

## Methods

### Ethics and registration

The study protocol was approved by the Clinical Research Ethical Committee of the First Affiliated Hospital of Soochow University (Approval No. 2024–411) on September 30, 2024. The study was registered at the Chinese Clinical Trial Registry^1^ (identifier: ChiCTR2400092927). Written informed consent will be obtained from all participants before inclusion in the study. This protocol adheres to the guidelines outlined in the Standard Protocol Items: Recommendations for Interventional Trials (SPIRIT) statement (17).

### Study design and participants

**Figure 1:**
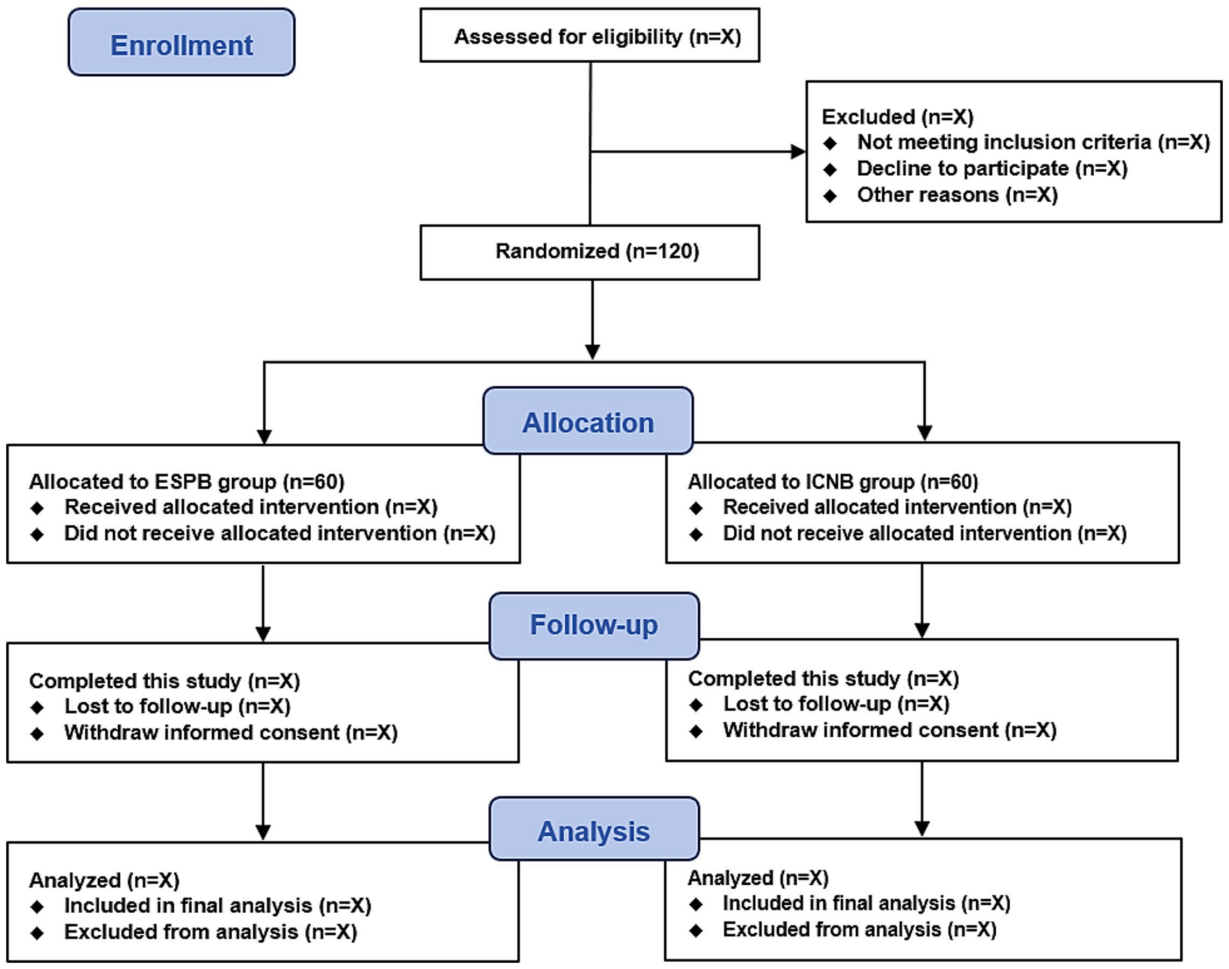
Study flow diagram. ESPB, erector spinae plane block; ICNB, intercostal nerve block.

This is a single-center, prospective, randomized, double-blinded clinical trial. This trial start date is 1 December 2024, with an anticipated completion date of 31 December 2025. The study flow diagram is presented in Figure 1. According to the SPIRIT statement, the patient recruitment, study interventions, and outcome measurement schedule are shown in Table 1.

**Table 1:** Schedule of patient enrollment, study interventions, and outcome measurements.

Timepoint	Study period								
	Enrollment	Allocation	Post-allocation						Close-out
	Preoperative visit	specimen removal	End of surgery	PACU	24 h after surgery	48 h after surgery	72 h after surgery	Hospital discharge	90 d after surgery
Enrollment									
Eligibility screening	**╳**								
Written informed consent	**╳**								
Demographic data	**╳**								
Randomization		**╳**							
Allocation		**╳**							
Interventions									
ESPB			**╳**						
ICNB			**╳**						
Measurements									
Pain scores at rest					**╳**	**╳**	**╳**		
Pain scores on activity					**╳**	**╳**	**╳**		
QoR-15 scores					**╳**	**╳**	**╳**		
First press on the PCIA device				**╳**	**╳**	**╳**			
Opioid consumption				**╳**	**╳**	**╳**	**╳**		
Initiate independent bedside mobilization					**╳**	**╳**	**╳**		
Postoperative hospital stay								**╳**	
Adverse events^a^				**╳**	**╳**	**╳**	**╳**		
Incidence of chronic pain									**╳**

According to SPIRIT statement of defining standard protocol items for clinical trials. PACU, post-anesthesia care unit; ESPB, erector spinae plane block; ICNB, intercostal nerve block; QoR-15, Quality of Recovery-15; PCIA, patient-controlled intravenous analgesia.

a Including postoperative nausea and vomiting (PONV), fever, headache, dizziness, itching, constipation, ileus, urinary retention, insomnia, new-onset atrial fibrillation, severe ventricular arrhythmia, cardiac arrest, chest tightness, pulmonary embolism, Pulmonary atelectasis, pneumothorax, chylothorax, Pneumothorax, deep vein thrombosis and organ failure.

Participants in this study should meet the following inclusion criteria: (1) American Society of Anesthesiologists (ASA) class I-III. (2) Aged between 18 and 75 years. (3) Scheduled for elective video-assisted thoracoscopic lung resection under general anesthesia. Participants will be excluded if they have any of the following conditions: (1) sinus bradycardia (heart rate < 50 bpm) or heart block (sinoatrial or atrioventricular block); (2) left ventricular ejection fraction <40%; (3) unstable coronary artery disease; (4) hepatic disease (Child-Pugh classification C) or renal failure (receiving renal replacement therapy); (5) diabetic neuropathy; (6) coagulation dysfunction; (7) infection at the puncture site; (8) history of Parkinson’s or Alzheimer’s disease; (9) history of epilepsy; (10) neurological disorders (e.g., stroke, hypoesthesia); (11) uncontrolled psychiatric disorders (e.g., anxiety, depression, schizophrenia); (12) pregnancy or breast feeding; (13) history of chronic pain or preoperative use of sedative and analgesic drugs; (14) allergy to local anesthetics or any trial-related medication.

### Randomization and blindness

An independent statistician, who will not participate in the study or data management, will generate a randomization list using an online tool^2^ with an allocation ratio of 1:1 and block sizes of 2 and 4. Randomization will be performed intraoperatively at the time of surgical specimen removal to minimize post-randomization exclusions. The generated random sequences will be securely sealed in sequentially numbered opaque envelopes stored in a locked cabinet. An independent researcher, who is responsible for patient enrollment, will immediately open the envelope following the removal of surgical specimen to obtain the randomization assignment. All patients will receive either an ESPB or ICNB based on randomization at the end of the surgery under anesthesia, ensuring that patients are blinded to their group allocations. Additionally, investigators involved in the postoperative outcomes data collection and follow-up assessments will remain masked to group allocation. Group allocation will remain concealed until the trial is closed to accrual and the final patient has completed the 3-month study period following surgery.

### Study interventions

The local anesthetic used for regional nerve block in both groups was liposomal bupivacaine (Bupivacaine Liposome Injection; Jiangsu Hengrui Pharmaceuticals Co., Ltd., China). For the ESPB group, a single erector spinae plane block is performed at the T5 vertebral level under ultrasound guidance by an independent anesthesiologist at the end of the surgery. The patient is positioned laterally, and a high-frequency linear ultrasound probe is placed 2–3 cm lateral to the T4 spinous process, then adjusted outward to locate the T5 transverse process in the sagittal plane. The erector spinae muscle is identified above the transverse processes. Under planar ultrasound visualization, the needle is carefully advanced until it contacts the bone of the T5 transverse process. Following the verification of proper needle position through a preliminary injection of 2 mL saline solution, 30 mL of local anesthetic (LB 266 mg mixed with 10 mL normal saline) is slowly administered after confirming the absence of blood or cerebrospinal fluid via aspiration.

For the ICNB group, an experienced surgeon identifies the T3 to T8 intercostal spaces under thoracoscopic visualization at the end of the operation. Subsequently, 3–4 mL of LB is injected into each intercostal space, approximately 3–5 cm lateral to the spinous processes at the T3 to T8 levels. The procedure is considered successful when pleural displacement is observed in all targeted intercostal spaces. In total, 20 mL of LB (266 mg) is administered across the intercostal spaces.

After surgery, all patients are provided with patient-controlled intravenous analgesia (PCIA), consisting of 100 μg sufentanil diluted to 100 mL with normal saline (final concentration: 1 μg/mL). The PCIA device is programmed with the following parameters: a background infusion rate of 1 mL/h, a self-controlled bolus of 2 mL, a lockout interval of 10 min, and a maximum hourly dose of 20 mL. The PCIA is initiated immediately after surgery and continues for 48 h postoperatively. If pain persists, defined as a numeric rating scale (NRS) pain score of ≥4, patients are instructed to self-administer sufentanil via PCIA. If pain remained unrelieved, intravenous rescue opioids (sufentanil, fentanyl, oxycodone, tramadol, or morphine) were permitted. Additionally, both groups will receive an initial intravenous dose of 50 mg flurbiprofen axetil at the end of surgery. This will be followed by two daily doses of 50 mg intravenous flurbiprofen axetil administered at 08:00 and 16:00 (totaling 100 mg per day) as part of the multimodal analgesia regimen on postoperative days 1 and 2.

### Perioperative management

Upon arrival in the operating room, patients receive standard monitoring, including non-invasive blood pressure measurement, electrocardiography, pulse oximetry (SpO_2_), and temperature monitoring. Following anesthesia induction, all patients undergo left or right radial artery catheterization for continuous arterial blood pressure monitoring. The anesthesia induction procedure follows a standardized protocol, involving the sequential administration of propofol (2–2.5 mg/kg), sufentanil (0.3–0.5 μg/kg), and cisatracurium (0.1–0.2 mg/kg). Endotracheal intubation is performed using a double-lumen tube for lung isolation, with proper positioning verified via fiberoptic bronchoscopy. The intraoperative mechanical ventilation protocol followed established lung-protective strategies (18). During one-lung ventilation, tidal volumes were set at 4–6 mL/kg, with an initial PEEP of 5 cmH₂O, followed by individualized adjustment. The inspired oxygen fraction and respiratory rate were modified to maintain SpO₂ ≥ 95% and end-tidal carbon dioxide (EtCO₂) levels between 35 and 45 mmHg during both one-lung and double-lung ventilation. Lung recruitment maneuvers were performed when clinically indicated. Anesthesia is maintained with sevoflurane inhalation, targeting a bispectral index value between 40 and 60. Intraoperative analgesia is provided with sufentanil and remifentanil: sufentanil 0.1–0.2 μg/kg is administered before surgical incision, and remifentanil (0.05–0.2 μg/kg/min) is continuously infused throughout the procedure. Sufentanil (0.1–0.2 μg/kg) is given when the remifentanil infusion is discontinued at the end of the operation. Incremental doses of cisatracurium are given intraoperatively as required.

Intraoperative hypotension (defined as a mean blood pressure [MBP] decrease of >30% from baseline or an MBP of <65 mmHg) would be treated with intravenous ephedrine of 6–10 mg or phenylephrine of 50–100 μg, and bradycardia (defined as heart rate [HR] of <50 beats/min) would be managed with intravenous atropine of 0.3–0.5 mg or ephedrine bolus if coexisting hypotension is present. Hypertension (defined as an MBP increase of >30% of baseline) will be treated with intravenous urapidil 5–10 mg. Tachycardia (defined as HR > 100 beats/min) will be treated with intravenous esmolol 10–20 mg.

At the end of the surgery, all patients receive ondansetron of 8 mg as an antiemetic after the final sutures are placed. Patients are then transferred to the post-anesthesia care unit (PACU) before being moved to the surgical ward. The Modified Aldrete Score is used to assess recovery in PACU, with a score of 9 or higher considered acceptable for discharge. To prevent any impact on pain perception, intravenous sedative drugs will not be administered after surgery.

### Data collection and registration

Trained independent investigators who are blinded to group assignment will collect the patients’ demographic data and follow-up outcomes.

Pain intensity is assessed using the numeric rating scale (NRS, 0–10; 0 = no pain, 10 = worst possible pain) at rest and during activity at 24, 48, and 72 h postoperatively, with a 2-h time window. Quality of Recovery-15 (QoR-15) scores are recorded at the same time points to evaluate overall recovery (19). This questionnaire measures five domains: pain, psychological state, emotional state, independence, and comfort. It consists of 15 statements rated on a 0–10 scale, with total scores ranging from 0 to 150. Higher scores indicate better postoperative recovery. Additionally, the time to the first press on PCIA device and the total amount of opioid consumption through 0–72 h postoperatively are documented. The time to first press on PCIA is measured in hours, calculated as the time of the first press on PCIA minus the time of the end of surgery. Total opioid consumption is standardized by converting all opioids to intravenous morphine milligram equivalents (MMEs) for each patient. For patients discharged before 72 h, follow-up assessments are conducted via telephone. The incidence of chronic pain (defined as NRS score ≥ 1) is evaluated through phone calls at 3 months postoperatively, with a permitted time window of 3 months ±7 days. Furthermore, the time to initiate independent bedside mobilization, and the length of the postoperative hospital stay are recorded. Independent bedside mobilization is defined as the patient being able to perform activities around the bed without assistance from others. The time to initiate independent bedside mobilization is measured in hours, i.e., the time of the event minus the time of the end of surgery.

All data will be entered into electronic case report forms (eCRFs) under the oversight of trained research personnel. An electronic trial database will be generated based on these eCRFs. Once data entry is finalized, the database will be locked and de-identified. The anonymized dataset will subsequently be transferred to an independent statistician for final analysis according to the prespecified statistical plan.

### Data monitoring committee

An independent Data Monitoring Committee (DMC) has been established to address any uncertainties related to data collection and registration. The DMC is composed of a chair (an experienced anesthesiologist), a professor of statistics, a pharmacologist, and a thoracic surgeon. If any ambiguity arises in data collection or registration, DMC will discuss these issues to reach a final consensus.

Both intervention methods are routinely performed at our center, and we anticipate that serious adverse events during this study will be infrequent. Any adverse event, whether related to the study interventions or not, must be reported to the DMC within 24 h using an “Adverse Event Form.” In the event of serious adverse events, such as unexpected deterioration of patients’ clinical status post-surgery, the research team could request unmasking of allocation and adjust or discontinue the study medications. The DMC will conduct ongoing reviews to assess safety and provide recommendations regarding the suspension or termination of the study.

### Study outcomes

The primary outcome of this trial is the average numeric rating scale (NRS) pain scores at rest over 72 h postoperatively (average of three 24-h time points: 24, 48, and 72 h). Secondary outcomes include NRS pain scores at rest and during activity at 24, 48, and 72 h postoperatively; Quality of Recovery 15 scores at 24, 48, and 72 h postoperatively; time to first press on the PCIA device; total amount of opioid consumption within 72 h postoperatively, converted to morphine milliequivalents; the time to initiate independent bedside mobilization, the length of the postoperative hospital stay and incidence of chronic pain (defined as an NRS score ≥ 1) at 3 months post-surgery. Safety outcomes included postoperative adverse events and complications within 72 h, including postoperative nausea and vomiting (PONV), fever, headache, dizziness, itching, constipation, ileus, urinary retention, insomnia, new-onset atrial fibrillation, severe ventricular arrhythmia, cardiac arrest, chest tightness, pulmonary embolism, pulmonary atelectasis, chylothorax, pneumothorax, deep vein thrombosis, and organ failure. The definitions of adverse events are displayed in Supplementary Table S1.

### Sample size calculation

Between March and June 2024, we conducted a prospective study in which patients received postoperative ultrasound-guided ESPB or ICNB under thoracoscopic visualization at the end of surgery using LB, with 10 patients per group. The results showed that the average NRS pain score at rest over the 72-h after surgery was 2.88 ± 1.75. The minimal clinically important difference (MCID) in pain scores for acute postoperative pain has been defined as 1 across a variety of surgeries (20–22). The sample size calculation was performed using a two-group t-test, assuming a standard deviation of 1.75, an 80% power, and a 0.05 level of significance. Considering a 15% dropout rate, the study will enroll a total of 120 patients, with 60 in each group. The sample size calculation was conducted using PASS software (version 15, PASS Institute Inc.).

### Statistical analysis

Descriptive statistics will be used to summarize baseline characteristics and demographic data without conducting between-group comparisons. Continuous data will be reported as median with interquartile ranges (IQR) or means with standard deviations (SD), depending on data distribution. Categorical data will be displayed as counts and percentages. The independent t-test or the Mann–Whitney U test will be used to assess between-group differences in continuous data, while the Chi-squared test or Fisher’s exact test will be applied for categorical data analysis, as appropriate.

For the study outcomes, the treatment effect of study interventions will be evaluated using odds ratios for binary data and differences for continuous data, each reported with 95% confidence intervals. The primary and secondary outcomes will be further analyzed using multivariable linear regression or logistic regression, adjusting for baseline covariates (age, sex, type of surgery, and number of ports). Subgroup analyses for the average pain score will be performed according to age (<60 years old vs. ≥ 60 years old), sex (female vs. male), BMI (< 25 vs. ≥ 25), surgery type (wedge resection vs. segmentectomy vs. lobectomy), and the number of port (single or multiple). Correction for multiple testing is planned for secondary outcomes using the Benjamini-Hochberg method.

The analysis will be performed on the modified intention-to-treat population, comprising all randomized patients who undergo VATS lung resection and for whom primary outcome are available. We do not plan to conduct missing data imputation or interim analysis. A Two-sided *p-*value of <0.05 will be considered statistically significant. Statistical analyses will be performed with R statistical software (version 4.3.0, R Foundation for Statistical Computing) by independent statisticians.

## Discussion

This prospective randomized controlled trial will recruit 120 adult patients undergoing elective VATS for lung resection. The study aims to compare the analgesic effects of LB administered via ultrasound-guided ESPB by anesthesiologists and ICNB performed under direct visualization by surgeons on the pain profile following VATS lung resection. Outcomes to be evaluated include NRS pain scores at rest and on movement at 24, 48, and 72 h postoperatively, QoR-15 scores at the same time points, time to first press on PCIA device, total opioids consumption within 72 h, time to initiate independent bedside mobilization, length of the postoperative hospital stay, incidence of chronic pain, and postoperative adverse events and complications. The primary hypothesis is that ultrasound-guided ESPB with LB will result in lower average pain scores at rest over 72 h compared to thoracoscopic ICNB with LB. Additionally, the trial will investigate adverse events associated with ultrasound-guided ESPB or thoracoscopic ICNB in the context of postoperative analgesia. This study will adhere to the Consolidated Standards of Reporting Trials guidelines.

Regional analgesia is a critical component of multimodal pain management and is highly recommended in VATS (23). The use of currently plain local anesthetics with a single dosage is restricted by the short duration of analgesia, typically less than 24 h. While continuous peripheral nerve blocks using a perineural catheter can extend the duration of pain relief, they are associated with potential drawbacks, including increased management complexity, risk of catheter-related infections, leakage, and accidental dislodgement (24). Liposomal bupivacaine is an innovative liposome-encapsulated local anesthetic designed for surgical site administration to yield long-acting postsurgical analgesia. Its distinctive multivesicular liposome structure forms a “medication depot,” allowing for sustained drug release through gradual membrane breakdown, thereby significantly extending the duration of analgesia (6, 25). Studies have shown that LB appears safe when used in fascial plane blocks and peripheral nerve blocks in VATS surgery, such as ESPB, paravertebral block (PVB), and intercostal nerve block (26–29).

The ESPB procedure, initially described by Forero et al. in 2016 for neuropathic pain management (12), has emerged as a promising alternative for pain relief, offering a more favorable safety profile. Notably, it is easy to perform due to the clear identification of anatomic landmarks on ultrasound and the absence of nearby vital structures. PVB has long been considered the gold standard for pain management in thoracotomy and VATS due to its well-established efficacy (30). However, PVB performs close to the spinal canal and vascular plexus, with technical complexity and potential risk of serious complications (31). In contrast, although the needle endpoint in ESPB is positioned farther from the paravertebral space and pleura, studies suggest that local anesthetic can still spread indirectly into the paravertebral space, providing analgesic benefits comparable to PVB while minimizing associated risks (32).

ICNB has been shown to provide effective pain relief after thoracic surgery and can be easily performed by surgeons during VATS (33). Recently, long-acting local anesthetics, such as LB, have been increasingly used for postoperative analgesia in ICNB during VATS lung resection. However, the efficacy of LB in controlling postoperative pain control following thoracoscopic procedures remains inconclusive. Several retrospective studies have suggested that LB is superior to bupivacaine hydrochloride for intercostal nerve blocks in managing postsurgical pain after thoracic surgery, including reducing postoperative opioid consumption and shortening hospital stays (34, 35). In contrast, two randomized controlled trials found that LB for intercostal nerve block did not offer superior analgesic or opioid-sparing benefits in the surgery (28, 36). Given that the analgesic effect of ICNB is often confined to a specific dermatomal distribution and requires multiple injections at each targeted intercostal space (10, 11), ESPB is easier to perform and provides a broader spread of local anesthetic, extending to paravertebral space, intercostal space, and neural foramina (37). Recent clinical practice increasingly favors ESPB over ICNB for postoperative management in thoracic surgery. A recent randomized controlled trial involving 60 consecutive adult patients undergoing mini-thoracotomy for lung resection indicated that ESPB provided superior analgesia, reduced perioperative analgesic consumption, and caused less respiratory muscle strength impairment compared to ICNB (15). Another randomized controlled study suggested that for minor pulmonary resection, where prolonged postoperative pain is not a concern, thoracoscopic ICNB may be beneficial due to its effectiveness in analgesia and procedural convenience. However, for more complex pulmonary surgeries associated with prolonged postoperative pain, ESPB appears to be a preferred option (16). These findings have predominantly relied on the use of plain local anesthetics. To date, this is the first randomized controlled study to compare the efficacy of anesthesiologist-performed ESPB and surgeon-administrated ICNB with LB in VATS lung resection, incorporating a comprehensive pain intensity assessment at 3 time points from 0 to 72 h postoperatively.

Our study has several limitations. First, we are unable to perform sensory testing to assess the effectiveness of the nerve blocks after surgery due to the intervention being conducted under general anesthesia. Second, pain assessments are conducted at three-time points during the first three postoperative days, with no additional time points evaluated beyond this period. Third, some patients are discharged within 72 h after surgery, necessitating follow-up of their NRS scores via telephone.

In conclusion, this randomized clinical trial is designed to evaluate the efficacy of anesthesiologist-performed ESPB using liposomal bupivacaine compared to surgeon-administrated ICNB with LB in VATS lung resection. We expect that LB can be safely employed in ultrasound-guided ESPB, and liposomal bupivacaine for anesthesiologist-performed ultrasound-guided ESPB will be more effective than surgeon-administrated thoracoscopic ICNB in reducing the average postoperative pain over the 72-h period.

## Footnotes

^1^ www.chictr.org.cn

^2^ https://www.sealedenvelope.com/simple-randomiser/v1/lists
